# Mesocolic hernia, a case series

**DOI:** 10.1016/j.ijscr.2024.109696

**Published:** 2024-04-25

**Authors:** Sayed Khedr, Sarah Magdy Abdelmohsen, Osama Abdelazim

**Affiliations:** a Lecturer of Pediatric Surgery, Aswan University Hospital, Aswan University, Egypt; b Lecturer of Pediatric Surgery at Cairo University Children Hospital, Cairo University, Egypt

**Keywords:** Internal hernia, Paraduodenal hernia, Intestinal obstruction, Single atrium, Meckel's diverticulum, Extrahepatic biliary atresia, BASM syndrome

## Abstract

**Introduction and importance:**

Paraduodenal hernias are difficult to diagnose due to their unusual presentation. Herein, five new cases are added to the literature.

**Case presentation:**

Four male and one female child complained of paraduodenal hernias, two on the right side and three on the left side. The intestinal part that herniated inside the hernia sac was also malrotated in four patients. One patient had Meckel's diverticulum with a herniated intestine. One infant had extrahepatic biliary disease, a single atrium, polysplenia, intestinal malrotation, and a left paraduodenal hernia. Exploratory labarotomy was done for reduction of the intestine, reorientation, and repair of hernia orifices.

**Clinical discussion:**

Paraduodenal hernia is a component of malrotation. Cautious dissection of the hernia orifice is required to keep away from injuries to the inferior mesenteric vein or left colic artery in the course of the restoration of the left paraduodenal hernia. Also, the superior mesenteric vessels may be injured in the course of the restoration of the right paraduodenal hernia.

**Conclusion:**

There is a correlation between the occurrence of PDH with malrotation. The diagnosis of malrotation can be made with an ultrasound abdomen; however, it is true that ultrasound cannot make a confirmed diagnosis in all patients. Once the diagnosis of a mesocolic hernia has occurred, surgical repair is mandatory by closure of the defect.

## Highlights

• Herein, five new cases of mesocolic hernias are adding to the knowledge.
• Some mesocolic hernias presented with other associated congenital anomalies.
• There is a correlation between the occurrence of PDH with malrotation.
• Once the diagnosis of a mesocolic hernia has occurred, surgical repair is mandatory by closure of the defect.

## 1 Introduction

An internal hernia is a circumstance wherein the belly organs or tissues go away from their authentic position and enter some other part of the abdomen via a vulnerable defect or area inside the belly cavity [[1], [2], [3]]. The occurrence of internal hernias ranges approximately from 0.6 % to 5.8 % [4,5]. The incidence of paraduodenal hernias (PDHs) is 53 % of all internal hernias [1,6,7]. It is more common in males than females and more predominant on the left side than the right side, in a ratio of 3:1 [1,[7], [8], [9]]. The correct diagnosis of PDH is rarely preoperative because of its unspecific symptoms and unspecific radiological investigation [1,10]. It is often asymptomatic. PDHs with clinical symptoms of intestinal obstruction are uncommon, accounting for about 0.2 % to 5.8 % of all PDHs. So, it needs a high degree of clinical suspicion [1,10,11]. Herein, a report of five patients who had PDHs was added to the knowledge base. This work has been reported in line with the PROCESS criteria [12].

## 2 Case reports

### 2.1 Case 1

A 2-year-old male child complained of recurrent attacks of colicky abdominal pain and recurrent attacks of alternative bilious and non-bilious vomiting for 2 days. There was no history of fever, blood in the stool, trauma, or previous operation, and the bowel habits were normal.

**Fig. 1:**
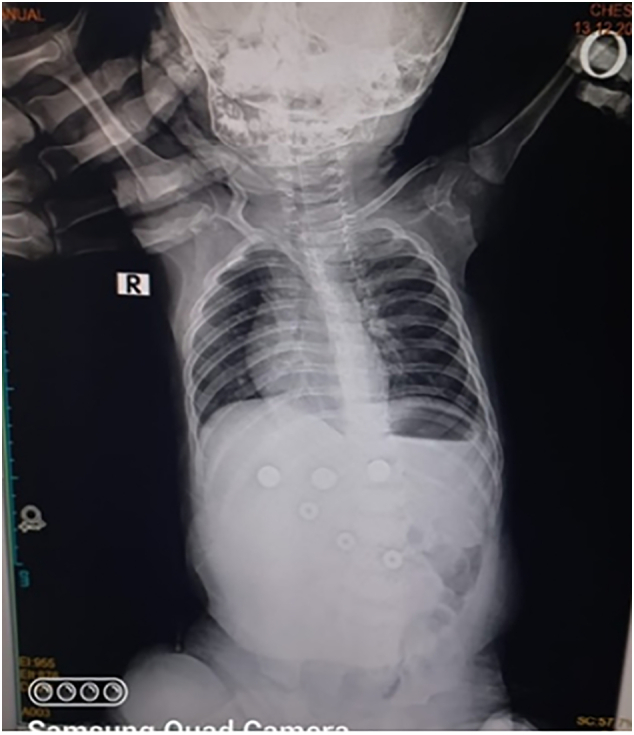
A plain erect abdominal X-ray revealed abnormal air distribution with scanty distal aeration.

Physical examination revealed a soft and lax abdomen with tenderness in the upper abdomen region. A plain erect abdominal X-ray showed abnormal air distribution with scanty distal aeration (Fig. 1). All blood investigations were normal except for moderate leukocytosis with neutrophil predominance. The child was admitted under observation for 24 h, but the gastric aspirate through the Ryle tube became bilious, and there was no improvement in the plain erect abdominal X-ray.

**Fig. 2:**
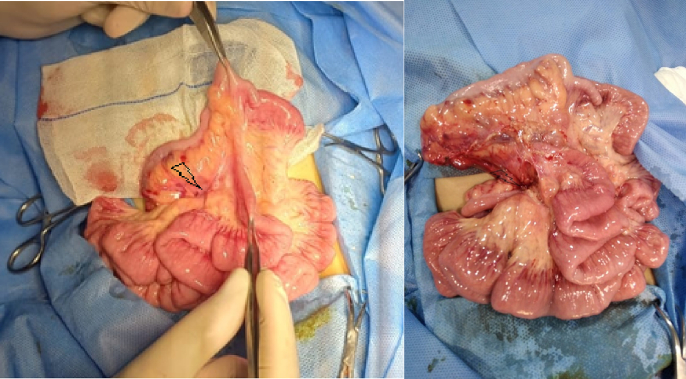
The black arrow showed the neck of the hernia sac, through which the small intestine herniated.

**Fig. 3:**
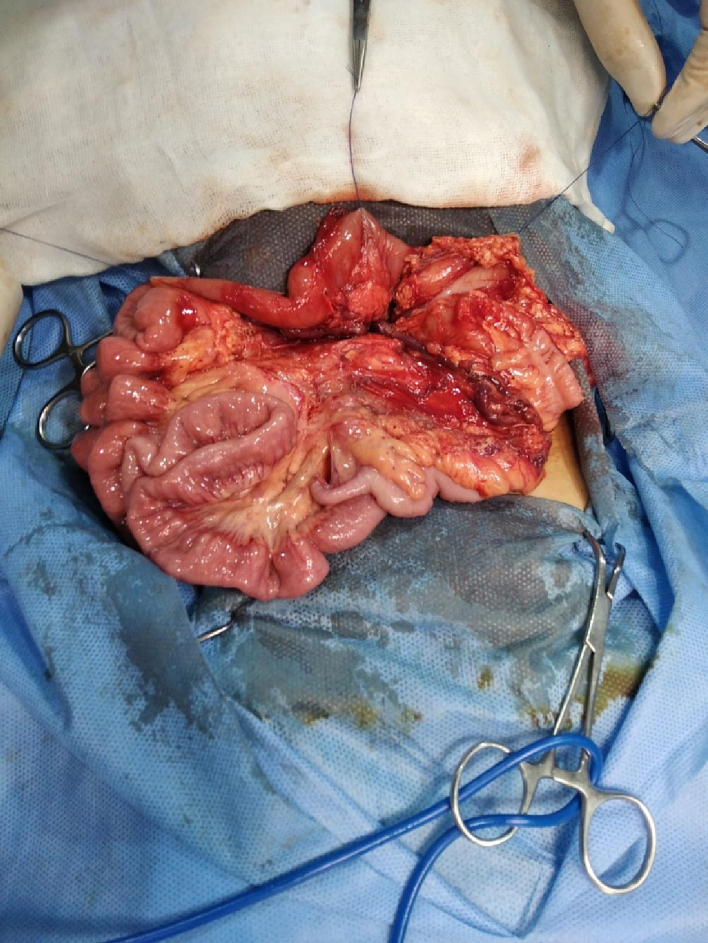
Closure of the hernia defect.

Exploratory laparotomy through a transverse supraumbilical abdominal incision demonstrated that the proximal 1 m of the jejunum was herniated through an opening of a hernia sac at the right side of the mesocolon associated with malrotation (right paraduodenal hernia or right mesocolic hernia) (Fig. 2). Reduction of the small intestine, which was fortunately healthy, and reorientation of the bowel were done (the small intestine was reoriented on the right side and the large intestine was reoriented on the left side). The neck of the hernia sac defect measured about 2 cm; closure of this defect was done by an experienced pediatric surgeon using interrupted stitches (Fig. 3). The postoperative period passed uneventfully. The patient was discharged after 5 days of full oral intake.

### 2.2 Case 2

A twelve-year-old male child had recurrent, intermittent attacks of colicky abdominal pain associated with non-bilious vomiting. The condition started when he was 8 years old and became more progressive during the last month. The condition is not associated with hematemesis, melena, bleeding per rectum, diarrhea, or fever. The child looks cachectic and has lost weight from 35 kg to 24 kg during the last two years.

**Fig. 4:**
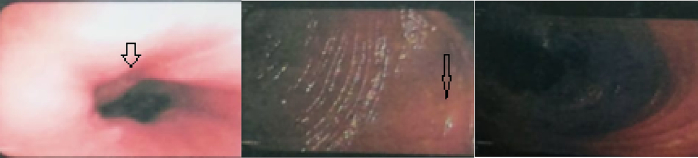
Upper GIT endoscopy reported a slightly inflamed cardia with an interrupted Z line. The stomach was filled with bile and had multiple patches of gastritis. The duodenum was dilated and had stunted haustrations.

**Fig. 5:**
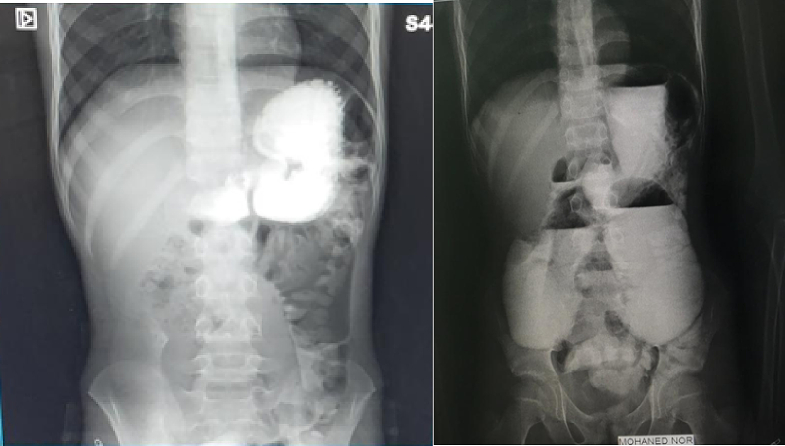
Post-contrast CT abdomen and pelvis revealed marked dilatation of the stomach, pylorus, and duodenum secondary to a short, tightly stenotic segment seen mostly at the duodenum-jejunal junction.

Abdominal examination revealed a soft, lax, non-tender, non-distended abdomen with visible peristalsis. Upper GIT endoscopy reported a slightly inflamed cardia with an interrupted Z line. The stomach was filled with bile and had multiple patches of gastritis. The pylorus was deformed and inflamed. The duodenum was markedly dilated with stunted haustrations (Fig. 4). Post-contrast CT of the abdomen and pelvis revealed marked dilatation of the stomach, pylorus, and duodenum secondary to a short, tightly stenotic segment seen mostly at the duodenum-jejunal junction. It was associated anteriorly with a whirlpool sign representing the clockwise rotating root of the mesentery and showing a few rotating vessels, a picture suggestive of a segmental midgut volvulus (Fig. 5).

**Fig. 6:**
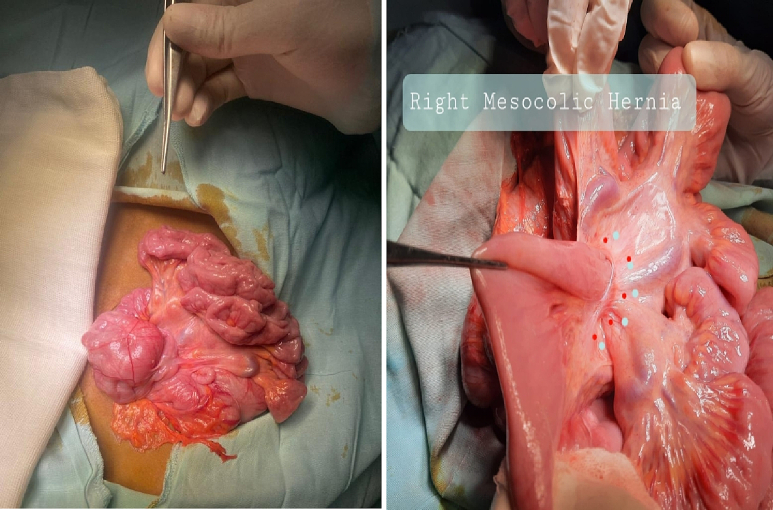
Right mesocolic hernia.

**Fig. 7:**
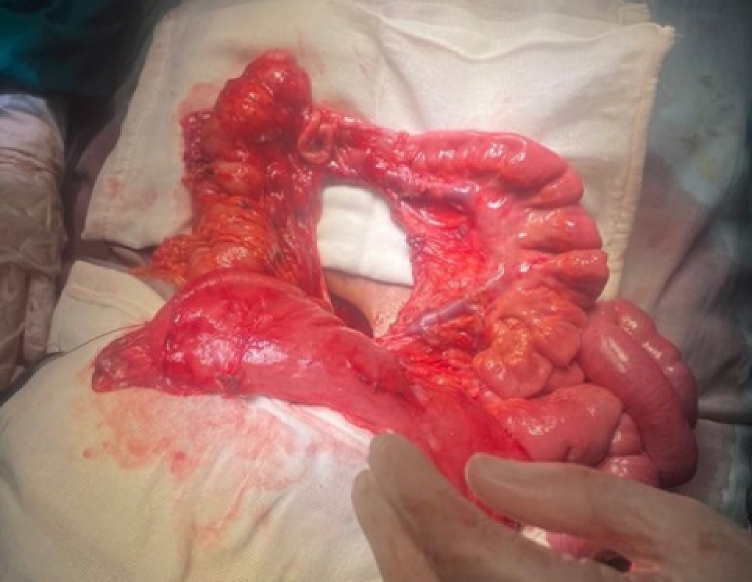
Hernia defect after small intestinal reduction and hernia sac excision. Also, the dilated duodenum and the appendix appear in the picture.

**Fig. 8:**
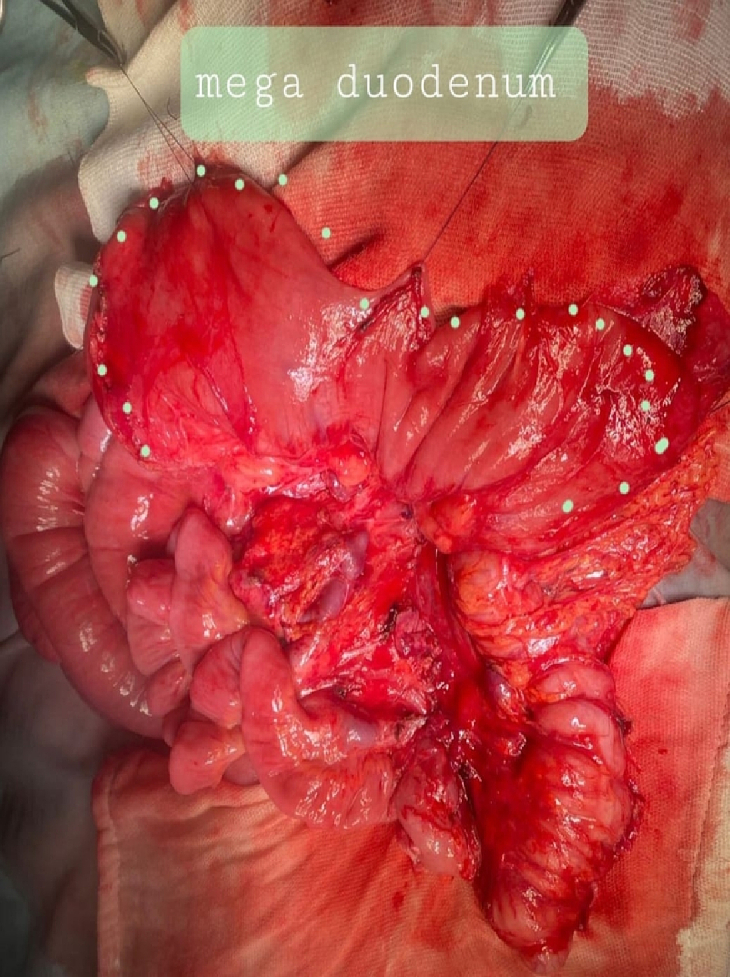
Marked dilated duodenum. This picture was taken after repair of the hernia defect.

The patient underwent an exploratory laparotomy with an upper midline incision. The patient had a right mesocolon hernia with a defect size of 3 cm in diameter (Fig. 6, Fig. 7). The sac's neck has been released. The duodenojejunal junction and the proximal 1 m of the jejunum were herniated through the defect. The duodenum was markedly dilated (Fig. 8). Reduction of the small intestine and reorientation were done (Fig. 7). Then excision of the sac, closure of the mesocolic defect with interrupted stitches, and appendectomy were done by pediatric surgeon. The postoperative period passed uneventfully, and the patient was discharged on the sixth postoperative day.

### 2.3 Case 3

A 3-year-old male child had a 2-day history of recurrent attacks of colicky abdominal pain, bilious vomiting, and constipation. The child had a history of 3 months' upper abdominal distension. There is no history of a previous abdominal operation, fever, diarrhea, hematemesis, or melaena. Physical examination revealed a distended abdomen with tenderness on the left side and an exaggerated intestinal sound. Immediately after Ryle tube insertion, the tube discharged 300 biliary fluids. Laboratory investigations were normal except for slightly elevated WBCs with neutrophil predominance.

**Fig. 9:**
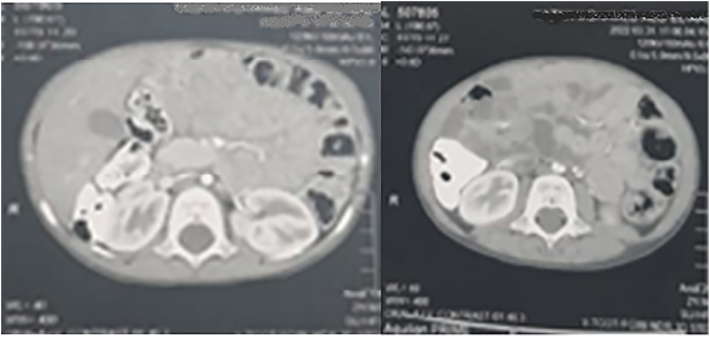
A cluster of small intestine loops was seen at the left anterior para-renal space on the postcontrast CT abdomen and pelvis axial plane.

**Fig. 10:**
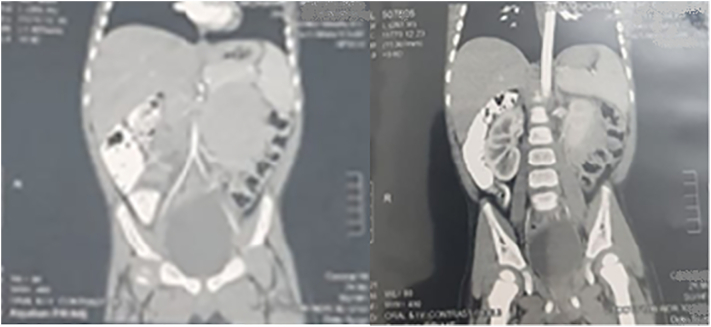
A cluster of small intestine loops were seen at the left anterior para-renal space on postcontrast CT abdomen and pelvis sagittal plane.

Abdominal ultrasonography revealed a dilated small intestine and an area of collected small intestine loop on the left side, indicating intussusception with minimal inter-looper collection. On postcontrast CT of the abdomen and pelvis, a cluster of small intestine loops was seen behind the inferior mesenteric vein in the left anterior para-renal space, indicating a left para-duodenal hernia (Fig. 9, Fig. 10).

**Fig. 11:**
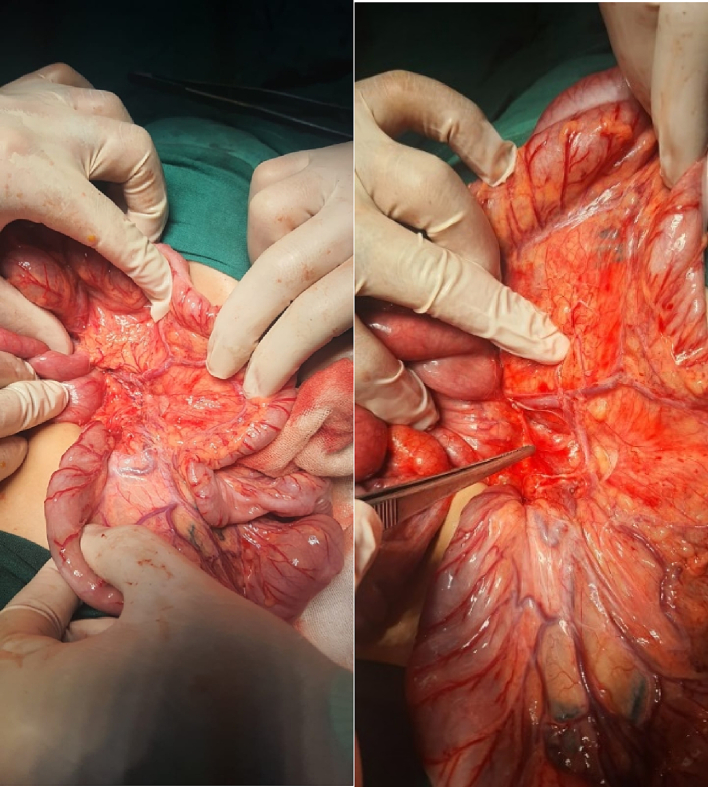
Left mesocolic hernia behind the level of the inferior mesenteric artery. The middle colic artery appears in this picture. The non-toothed forceps are attached to the edge of the hernia defect.

**Fig. 12:**
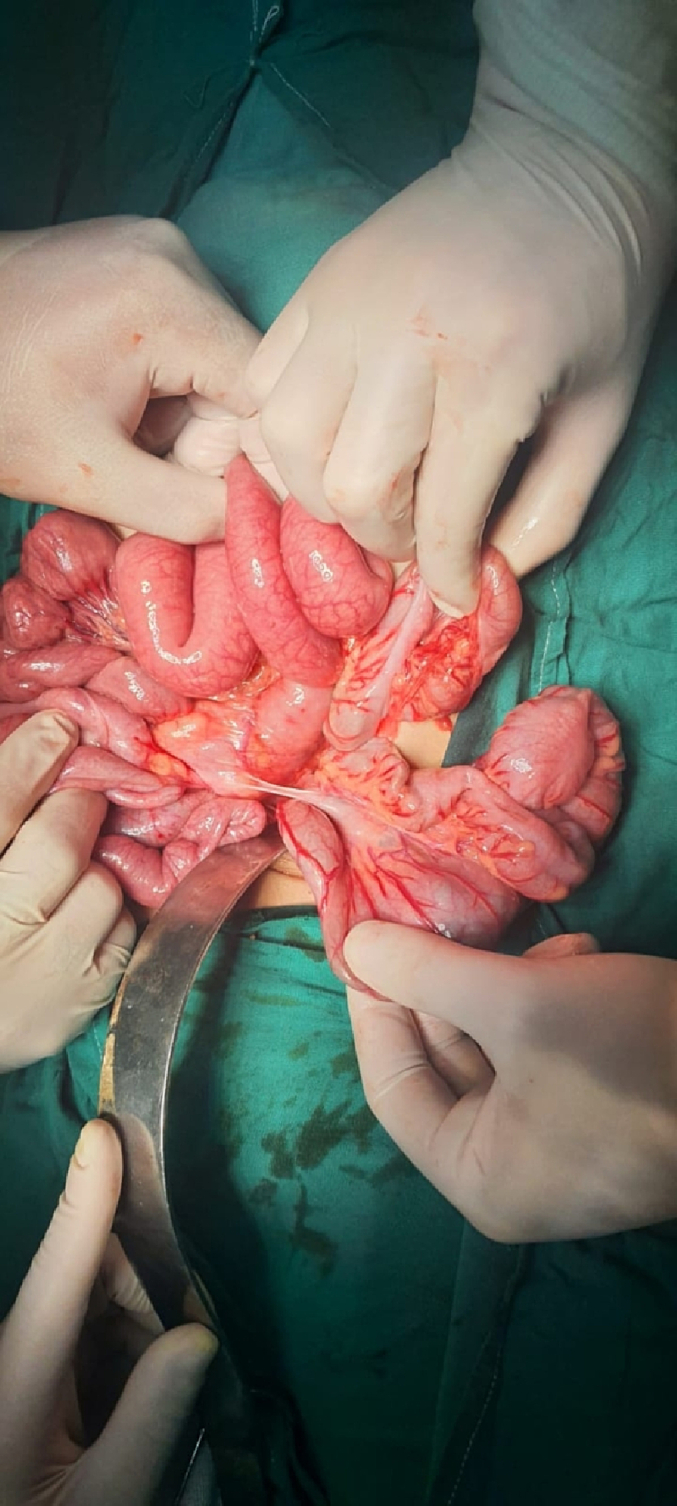
Meckel's diverticulum with adhesive band from its tip to adjacent area of the jejunum.

**Fig. 13:**
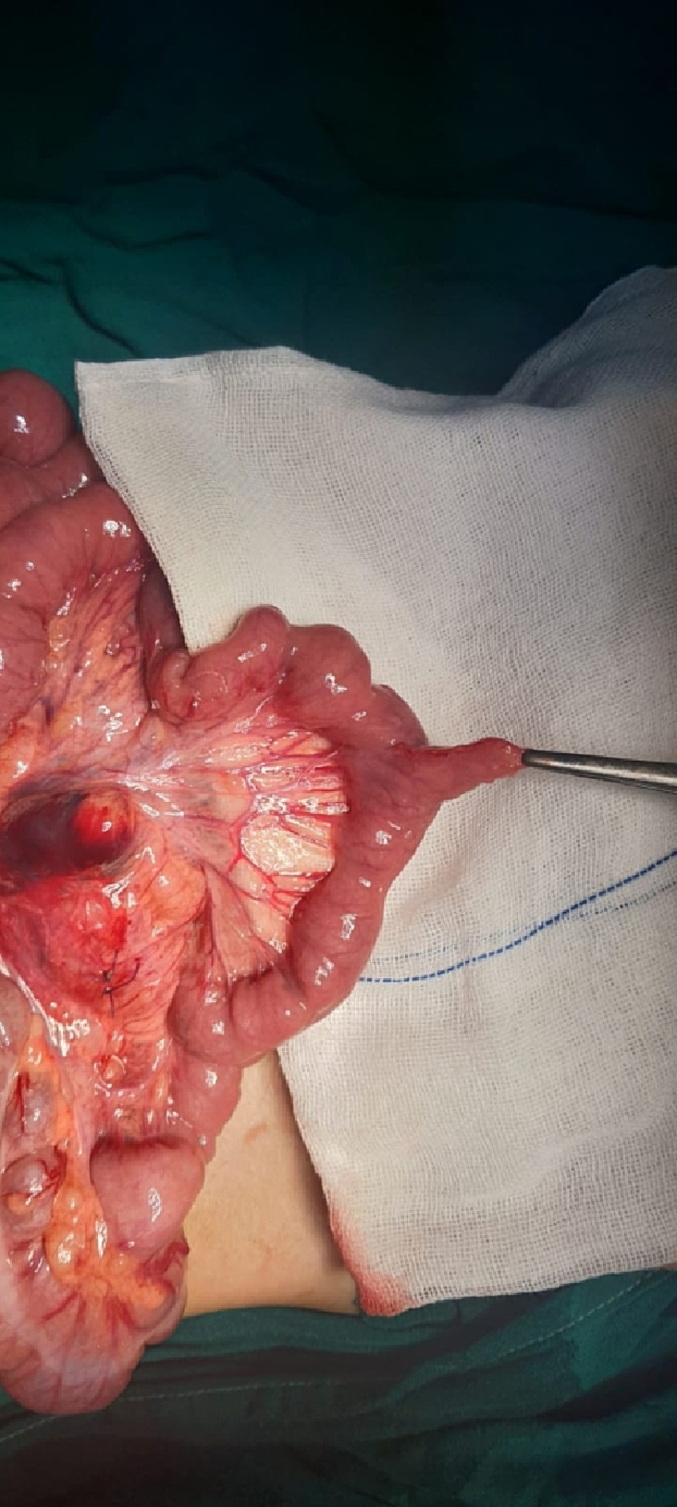
After adhesiolysis to the adhesive band.

The patient underwent an urgent exploratory laparotomy through the upper midline abdominal incision. The intestinal loops were herniated through a potential space on the left side of the Treitz ligament, between the duodenojejunal junction and transverse mesocolon, behind the inferior mesenteric vein (left mesocolic hernia) (Fig. 11). The size of the hernia defect was 2 cm. After reduction of the small intestine, Meckel's diverticulum with an adhesive band from its tip to the adjacent area of the jejunum and multiple mesenteric LNs appeared (Fig. 12). The small intestine was healthy. Adhesiolysis of the adhesive band and wedge excision of the Meckel's diverticulum were performed, followed by transverse ileal loop repair with interrupted sutures (Fig. 13). Repair of the defect with Vicyrl 3-0 was done. The patient started oral feeding on the 2nd postoperative day and was discharged on the 5th postoperative day with a good outcome.

### 2.4 Case 4

A 11-year-old male child complained of recurrent attacks of colicky abdominal pain of 20 days' duration. Over the last two days, the pain became exaggerated and was associated with non-bilious vomiting. For two years, the child had experienced recurrent episodes of abdominal distension that resolved on their own. Also, the child had the same attack of colicky abdominal pain two years ago, which resolved spontaneously. There were no other symptoms like fever, melena, diarrhea, or hematemesis.

An abdominal examination revealed right lower abdominal tenderness associated with positive Cope's and Psoas signs. The total leucocytic count was slightly elevated, with neutrophils predominating. Abdominal ultrasonography reported a 1 [6–].cm-long blind-ended tubular structure with minimal free intraperitoneal collection on the right lower side of the abdomen; a picture suggested of acute appendicitis.

**Fig. 14:**
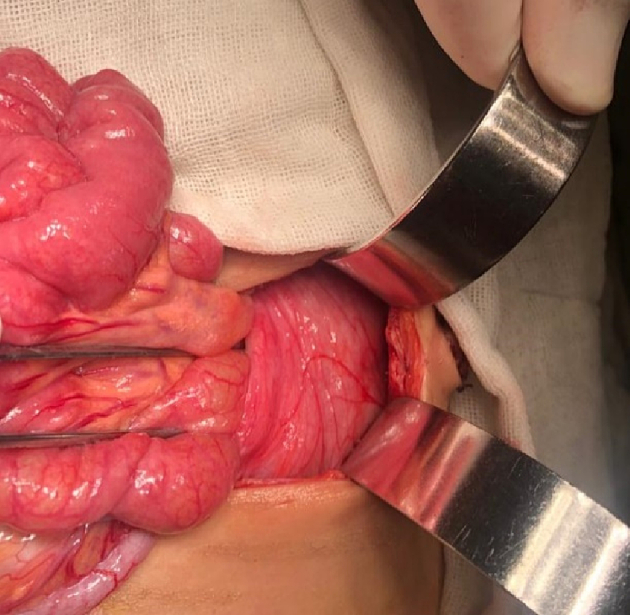
Left mesocolic hernia.

**Fig. 15:**
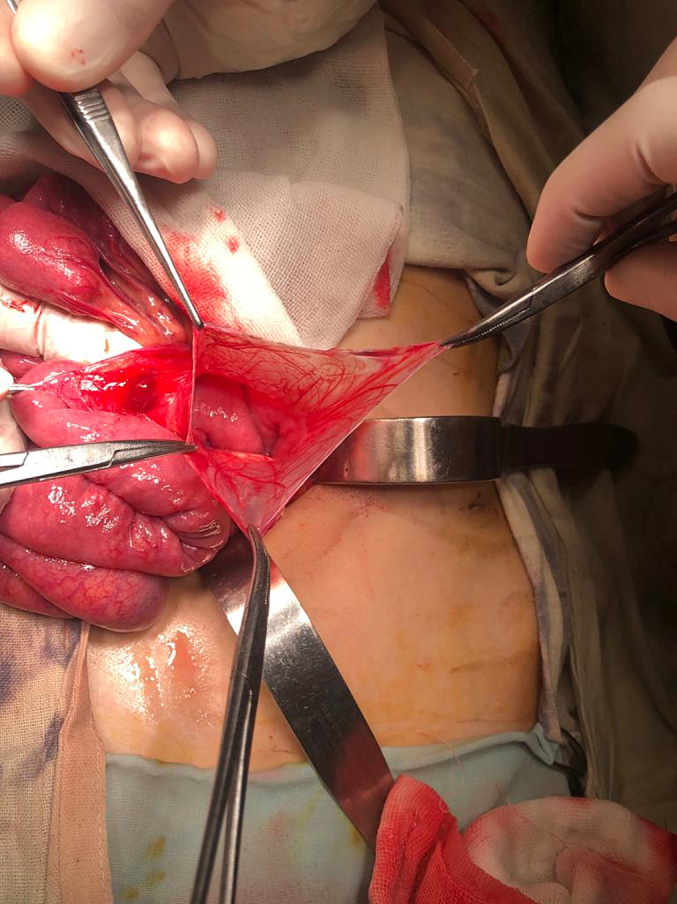
The hernia sac orifice.

**Fig. 16:**
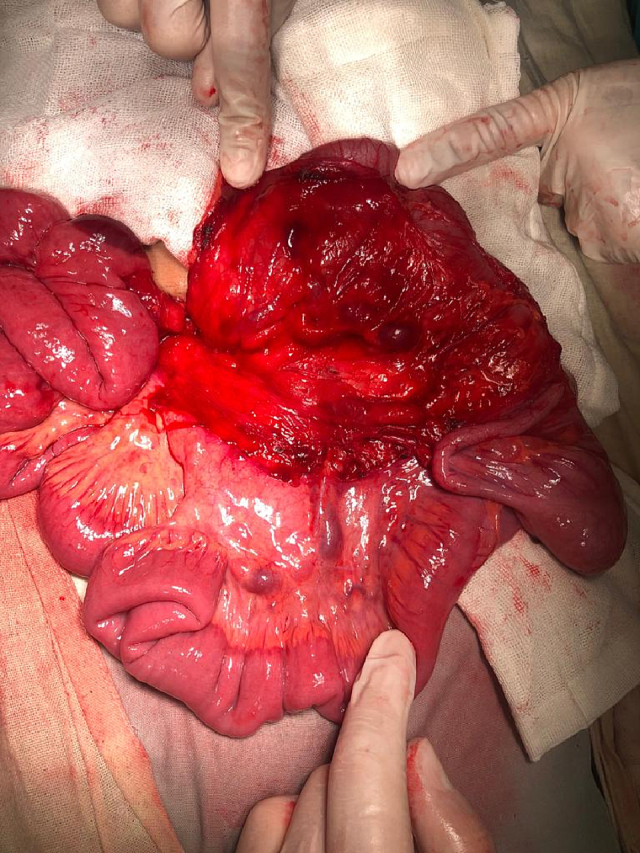
The herniated intestine was congested.

The patient was exposed to operative intervention using a transverse Lanz incision. On exploration, the caecum was not detected. The Lanz incision extended one and a half cm to the left side. The surgeon detected intestinal malrotation with herniation of part of the small intestine through a hernia sac behind the left mesocolon (left mesocolic hernia) (Fig. 14) (Video 1). The size of the hernia neck orifice was 3 cm (Fig. 15). After reduction of the congested small intestine, the color returned to normal after 1 min, and the appendix appeared normal (Fig. 16). Closure of the hernia sac with interrupted stitches and repositioning of the gut were done (small gut on the right side and massive gut on the left). The patient started oral fluids on the 2nd postoperative day and was discharged on the 4th postoperative day with a good outcome.

### 2.5 Case 5

A 70-day-old female infant presented with jaundice and clay-colored stool 3 weeks after birth. A full-term infant was born by caesarian section. She was the third child of a non-consanguineous marriage. There was no history of maternal disease, drug, radiological, or any other risk factor exposure.

Laboratory investigation revealed that total bilirubin was 11 mg/dl, direct bilirubin was 9 mg/dl, gamma-glutamyl transferase (GGT) was 688 U/LU/l alanine transaminase (ALT) was 156, and aspartate transaminase (AST) was 274. It is a laboratory picture suggestive of cholestasis.

**Fig. 17:**
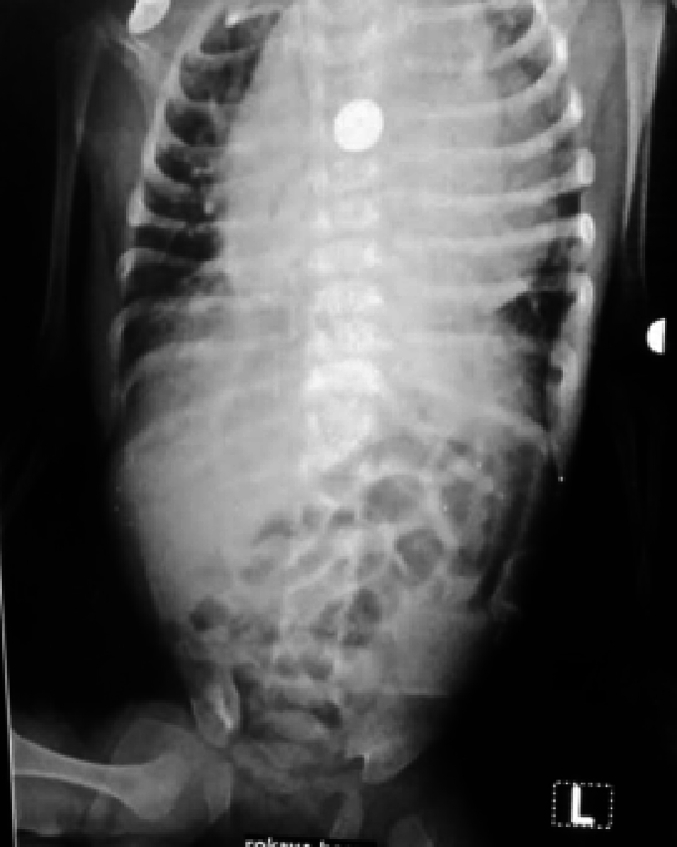
Preoperative echocardiography showed cardiomegaly.

Abdominal ultrasonography showed an absent gall bladder, a triangular cord sign in the liver hilum suggesting extrahepatic biliary atresia, and polysplenia. A liver biopsy reported marked liver fibrosis, cholestasis, and bile ductulus proliferation, a picture suggested extrahepatic biliary atresia. Echocardiography reported a single dilated atrium, a small inlet ventricular septal defect measuring 4 mm with a bidirectional shunt mainly left to right, pulmonary valve stenosis, and pulmonary hypertension of 75 mmHg (Fig. 17).

**Fig. 18:**
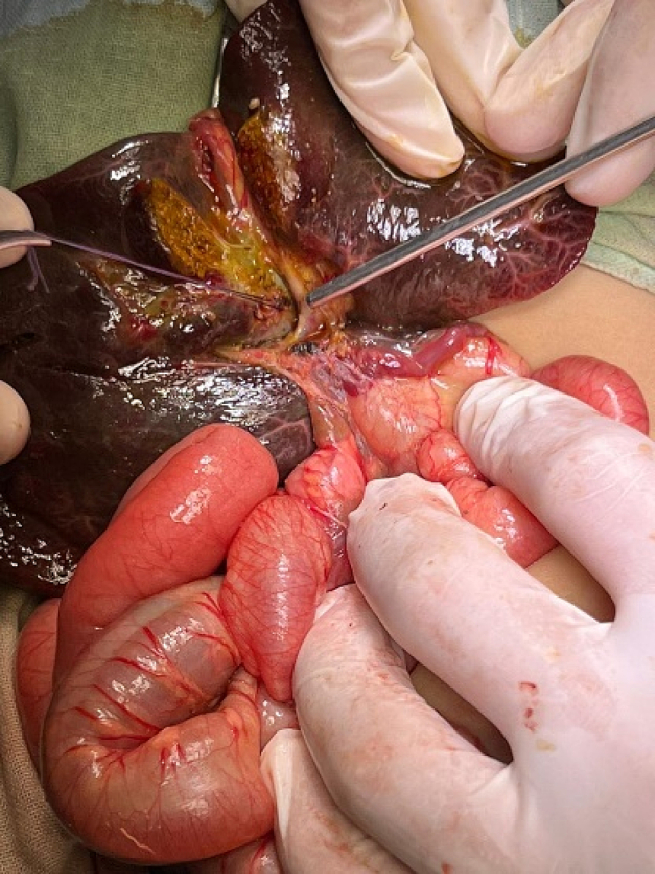
Extrahepatic biliary atresia.

**Fig. 19:**
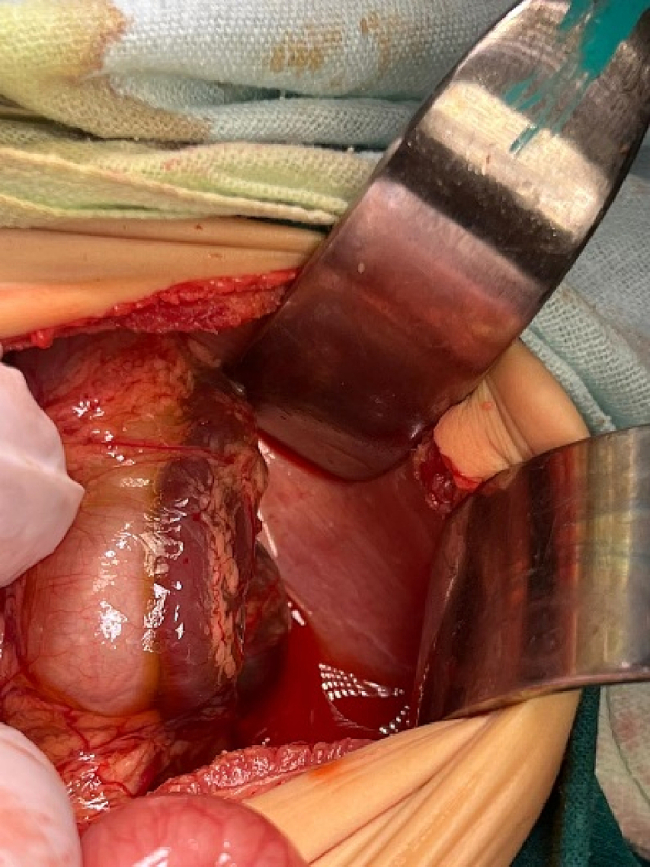
Polysplenia.

**Fig. 20:**
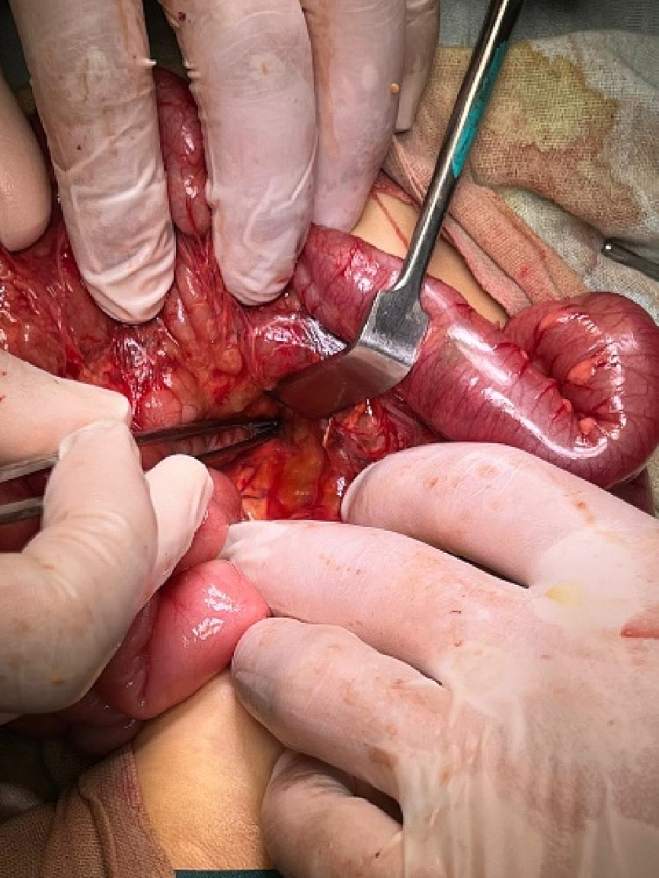
The defect of left paraduodenal hernia.

**Fig. 21:**
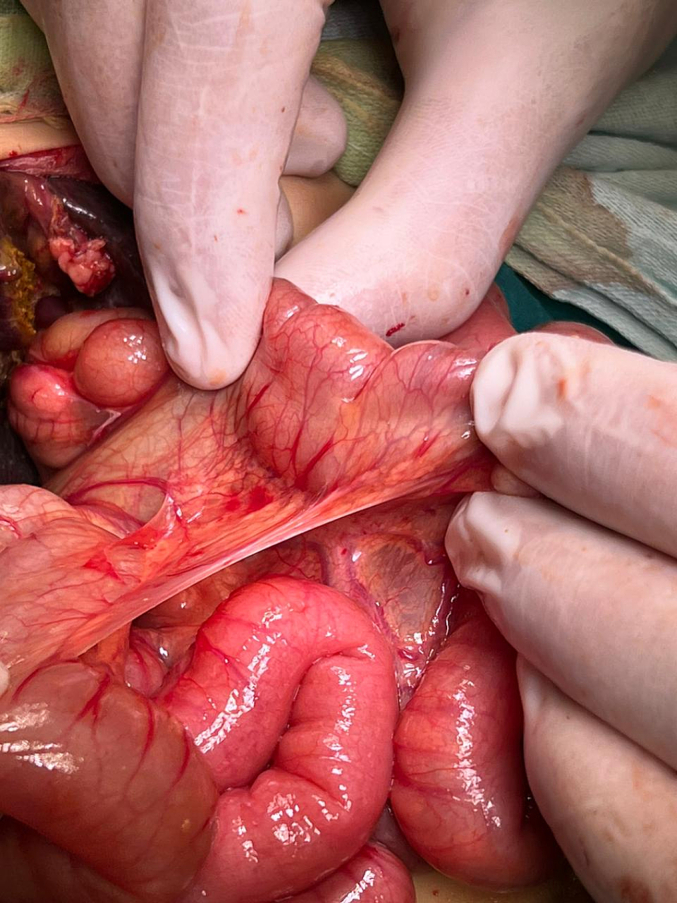
Ladd's band of intestinal malrotation.

A 2 mg vitamin K IM injection at least 24 h before surgery and perioperative antibiotic prophylaxis in the form of cefazolin at a dose of 30 mg kg IV within 30 min before surgery were administered to the infant. A right subcostal oblique incision with muscle cutting and liver mobilization was done. Intra-operative findings were atretic gall bladder, extrahepatic biliary atresia type III (Fig. 18), polysplenia (Fig. 19), left PDH (Fig. 20), and intestinal malrotation (Fig. 21). The left PDH discovered incidentally during the operative intervention. An experienced pediatric surgeon had reduced the small intestine from the hernia defect and closed it with Vicryl 4-0. Correction of malrotation used the lad's procedure. Then Kasai portoenterostomy and Roux en Y jejunojejunostomy by Vicryl 4-0.

The patient was kept in ICU, started oral feeding on the 3rd postoperative day, and passed colored stool on the 4th day. Unfortunately, the infant died on the 14th postoperative day due to heart failure.

## 3 Discussion

PDH was first defined by Treitz in 1857 and then by Moynihan in 1906 [1,6,13,14]. The etiology of PDH is unclear [7]. It can be congenital or acquired. Congenital orifices encompass an ordinary foramen like foramen of Winslow or uncommon peritoneal fossae or recesses arising from anomalies of internal rotation and peritoneal attachment [3,15]. It was created due to the failure of complete intestinal rotation during the 10th and 11th weeks of gestation, which results in the failure of peritoneal fusion [3,7,14]. Whereas acquired orifices result from trauma, inflammation, or previous surgery [3,15].

2 % of individuals in autopsies have a fossa of Landzert, and they are more susceptible to affection with the left PDH [1,16]. 1 % of individuals have a fossa of Waldeyer, and they are more affected by the right PDH [1]. The fossa of Landzert is an untypical congenital peritoneal fossa in the back of the descending mesocolon. The inferior mesenteric vein and ascending left colic artery are landmarks located on the anteromedial border of the fossa of Landzert (Video 1), while the duodenojejunal junction is on the alternative border [3]. The small opening in Waldeyer's fossa is bordered by the third part of the duodenum, the superior mesenteric vessels, and the posterior peritoneum [7,17,18].

Clinically, these patients often present with postprandial pain, which is typically chronic in nature and has symptoms dating back to childhood. On radiography signs, PDHs present as an encapsulated, circumscribed mass of a few loops of small bowel in the left upper quadrant, lateral to the ascending duodenum. These loops may have a mass effect, depressing the distal transverse colon and indenting the posterior wall of the stomach [15]. Once the diagnosis of PDH has been made, the repair should be done. When repairing left PDHs, take care not to injure the inferior mesenteric vein or left colic artery. Also, take care when repairing the right PDHs for the superior mesenteric vessels. The superior mesenteric artery supplies most of the small intestine and ascending colon, so its injury is a catastrophe [5,19]. We believe that an open surgical exploration in these cases is better than laparoscopic intervention regarding visualization and more easy handling of the intestine.

Case No. 5 is an interesting case. It's a picture of BASM syndrome (biliary atresia with polysplenia) [20]. However, this case is more unique because of its associated cardiac anomalies, intestinal malrotation, and left PDHs. Also, the presence of Meckel's diverticulum in Case No. 3 makes it a special case report.

## 4 Conclusion

The surgeon should keep in mind that there can be a possibility of PDH in a patients suspected of having malrotation. The diagnosis of PDHs may be delayed due to unusual symptoms, but once the diagnosis has occurred, surgical repair should be done as soon as possible by closure of the defect. Some PDHs presented with other associated congenital anomalies.

The following is the supplementary data related to this article.

Video 1 Video 1

## Consent

Written informed consent was obtained from the patients' parents for publication of this case series and accompanying images. A copy of the written consent is available for review by the Editor-in-Chief of this journal on request.

## Ethical approval

Approved by Aswan university medical ethical committee no. 899/2/24.

## Funding

No funding.

## Author contribution

**SMA** Wrote the manuscript, editing, study design, analysis, surgeon for one case and follow up of the patients.

**SK** Data collection, the surgeon and follow up of the patients.

**OA** Data collection, the surgeon and follow up of the patients.

## Guarantor

Sarah Magdy Abdelmohsen.

## Research registration number

No.

## Conflict of interest statement

No conflict of interest.

## Data Availability

Available when editor requests.
